# Potential Application of Resistant Starch Sorghum in Gluten-Free Pasta: Nutritional, Structural and Sensory Evaluations

**DOI:** 10.3390/foods10050908

**Published:** 2021-04-21

**Authors:** Mariasole Cervini, Alice Gruppi, Andrea Bassani, Giorgia Spigno, Gianluca Giuberti

**Affiliations:** 1 Department of Biotechnology, University of Verona, Strada Le Grazie 15, 37134 Verona, Italy; mariasole.cervini@univr.it; 2 Department for Sustainable Food Process (DiSTAS), Università Cattolica del Sacro Cuore, via Emilia Parmense 84, 29122 Piacenza, Italy; alice.gruppi@unicatt.it (A.G.); andrea.bassani@unicatt.it (A.B.); giorgia.spigno@unicatt.it (G.S.)

**Keywords:** resistant starch, hydrolysis index, dietary fibre, pasta, annealing

## Abstract

Gluten-free (GF) pasta samples containing rice flour replaced with 0, 5, 10, 15 g/100 g (*w*/*w*) of a resistant starch ingredient from annealed sorghum starch (annRS) were formulated. The highest total dietary fiber and RS contents (*p* < 0.05) were measured in uncooked pasta with 15 g/100 g of annRS addition (15-annRS). After cooking, the 15-annRS pasta was characterized by an RS content of 5.8 g/100 g dry matter, confirming the thermal resistance of annRS. The use of annRS positively influenced the optimal cooking time, the cooking loss, the firmness, and the stickiness of the cooked samples, with not remarkably change in color after cooking. The starch hydrolysis index values decreased as the level of annRS increased. Despite a significant decrease in the overall sensory with increasing levels of annRS, all samples were characterized by a value > 5, which is considered the limit of acceptability. The use of annRS in GF pasta up to 15 g/100 g can contribute to creating GF products with high total dietary fiber content, slowly digestible starch properties, and without drastically compromising the sensory attributes.

## 1. Introduction

Cereal-based gluten-free (GF) products are not only exclusively consumed by individuals suffering from medically diagnosed coeliac disease but by a growing number of consumers who spontaneously reduce and/or avoid gluten from their eating habits [1]. However, divergences regarding the nutritional quality of cereal-based GF foods compared to gluten-containing counterparts are still present [2]. In particular, data comparison of the nutritional composition of cereal-based GF alternatives to gluten-containing foods generally indicates lower dietary fiber content, higher glycaemic index, and higher total fat content [3,4,5]. The nutritional imbalance of GF cereal-based products may also contribute to weight gain and related metabolic diseases for individuals following a strict GF diet [6,7]. Starting from these considerations, research has been conducted to ameliorate the nutritional profile of different cereal-based GF products, including dry pasta.

Overall, dry pasta is considered a suitable product to reformulate as GF, aiming to improve the nutritional profile [8]. In this context, one of the most flexible strategies is the partial replacement of common GF flours and/or starches with novel nutrition-dense ingredients [1,2,8,9]. In regards to raw materials, the potential use of ingredients rich in resistant starch (RS) is gaining importance in making GF pasta [5,10,11,12].

The RS fraction is that fraction of starch that escapes digestion in the small intestine to be fermented in the large intestine favoring a series of health-related benefits comparable to those of dietary fiber [13,14]. Besides, increasing the RS amount in pasta may result in lower glycaemic carbohydrate content and lower in vitro starch digestibility [15], even if discrepancies exist, probably related to the type and properties of the RS used in the formulation [10,11,15,16]. In this sense, as a function of the inherent heat stability of the RS, the pasta manufacturing process along with the cooking step can destroy most forms of RS [11,17,18]. This gives importance to the search for ingredients containing thermally stable forms of RS and to the need to evaluate their functionality and effect on pasta formulation.

Several attempts have been made to generate heat-stable RS ingredients through physical, chemical, and enzymatic treatments of different native starches [19,20]. Besides, there is a need to find alternative underutilized RS sources for possible food applications. In this context, promising results have been reported by subjecting isolated white sorghum starch to annealing (annRS) [19]. The authors reported that the resulting annRS had a high RS content and greater heat stability to the native starch form. This novel RS-rich ingredient has been so far tested in GF biscuit formulation [21]. In particular, the use of annRS up to 45 g/100 g in the recipe contributed to the formulation of products with high RS content, slowly digestible starch properties, and without compromised quality and sensory attributes [21]. However, to the best of our knowledge, information concerning its functionality in GF dry pasta is lacking.

To better explore the potential of the annRS in GF dry pasta, GF pasta was formulated by replacing rice flour with increasing levels of annRS (up to 15 g/100 g) in the recipe. Newly developed products were evaluated for their RS content prior to and after cooking, along with the in vitro starch digestibility on cooked samples. Sensory analysis was also conducted to explore if the use of annRS could play a role in modifying the sensory attributes.

## 2. Materials and Methods

### 2.1. Raw Materials

White rice flour was supplied from Pedon S.p.A. (Molvena, Italy). As reported in the label, the chemical composition was moisture content 7.7 g/100 g; crude lipid 1.6 g/100 g; total starch 75.9 g/100 g; total sugar 0.5 g/100 g; crude protein 7.1 g/100 g, total dietary fiber 1.5 g/100 g of product. The particle size of the rice flour was <0.2 mm. The annRS ingredient was obtained from annealed white sorghum starch as previously detailed [19]. White sorghum starch was firstly isolated from commercial dehulled white sorghum flour ((*Sorghum bicolor* (L.) Moench)) purchased from CiboCrudo s.r.l. (Roma, Italy) and then dispersed in distilled water (ratio of 1:4 *w*/*v* starch to water) at 50 °C for 24 h under constant agitation. The liquid fraction was removed after centrifugation (4000 rpm; 15 min), and the remaining solid residue containing the annRS was oven-dried at 40 °C for 12 h (final moisture content of 8.7 g/100 g) and finely ground (0.5 mm screen; Retsch ZM1; Brinkman Instruments, Rexdale, ON, Canada). The RS content of the annRS was 53.5 g/100 g dry matter (DM), whereas the RS of white rice flour was 1.7 g/100 g DM.

### 2.2. Pasta Preparation

Macaroni-shaped GF pasta was produced in a customized plant installation (about 12 kg/h) consisting of a mixer, an extruder, and a cabinet dryer. The control GF pasta recipe contained rice flour (99.5 g/100 g dry flour basis) and mono- and di-glycerides of fatty acids (0.5 g/100 g; E471; Lucgel S.r.L, Perugia, Italy) (control). The annRS-enriched blends containing 5, 10, and 15 g/100 g *w*/*w* annRS were produced by replacing rice flour with the corresponding annRS level (5-annRS, 10-annRS, and 15-annRS, respectively). For each formulation, dry flour blends (6 kg) and tap 37 °C water were mixed (13 min; Procut Omni20, Inox-Fer s.r.l., Reggio Emilia, Italy) to obtain a uniform hydrated mass with a final water content of 35 g/100 g. The hydrated mass was heated in the mixer by steam at 0.3 MPa at 120 °C for 15 min to induce starch gelatinization. Then, it was formed in a single-screw extruder with a bronze macaroni-shaped die under vacuum conditions (La Parmigiana model RZ50, Parma, Italy) by keeping the dough temperature < 50 °C. The auger extrusion speed was 20 rpm. Samples were dried at 50 °C for 14 h in a cabinet dryer (La Parmigiana model ESS20, Parma, Italy). The control pasta was prepared under the same conditions. Dried GF pasta samples were stored at room temperature until analyzed. For each recipe, two batches were produced. The highest level of inclusion of annRS in the recipe was selected considering preliminary trials. Going beyond this level caused difficulties associated with the extrusion process.

### 2.3. Chemical Composition

Dry pasta was analyzed for proximate composition, including DM, ash, crude protein, crude lipid, and total starch [22]. The total dietary fiber (TDF) content was assessed enzymatically (Megazyme assay kit K-INTDF 02/15). This assay kit includes RS in the assessment of the TDF content in foods. A commercial assay kit (K-RSTAR 02/17, Megazyme International, Wicklow, Ireland) was used for the quantification of RS in both uncooked and cooked samples following manufacturer instructions. For cooked samples, 50 g of GF pasta were boiled in distilled water to optimal cooking time (OCT; see the specific paragraph), treated with liquid nitrogen, and lyophilized (method 2002.02) [22]. Samples were ground through a 0.5-mm screen. The apparent RS retention (aRSr) was calculated as follows:

aRSr = RS in cooked sample (g/100 g dry weight)/RS in uncooked sample (g/100 g dry weight) × 100(1)

### 2.4. Color Evaluation

The surface color of uncooked and cooked samples was measured through a Minolta CR410 Chroma Meter (Konica Minolta Co., Tokyo, Japan). The CIELAB system color space (L*, a*, and b*) was considered. The D65 standard illuminant and a visual angle of 10 were used. Five readings were taken for each sample.

The total color difference (ΔE*) were calculated as follows:

ΔE*_s−c_ = [(L*_s_ − L*_c_)^2^ + (a*_s_ − a*_c_)^2^ + (b*_s_ − b*_c_)^2^]^1/2^ (2)

where: s = annRS containing pasta and c = control. The ΔE* value > 3 indicates whether the color difference was perceivable by the human eye [23]. Before measuring, cooked pasta was carefully dried with absorbent paper.

### 2.5. Pasta Quality

The OCT was determined with the AACC-approved method 66-50 [24]. The cooking loss was determined by evaporating the cooking water to dryness at 105 °C (method 66-50) [24]. The water absorption capacity (WAC) was determined with the method AACC 66-50 [24]. Briefly, 25 g of pasta was cooked in 300 mL of boiling distilled water, rinsed in cold water, drained for 30 s, and weighed. The WAC was calculated as the relative weight increase after cooking.

### 2.6. Texture Properties

Texture characteristics (AACC method 66-50) [24] were conducted with a TA-XT2i Texture Analyser (Stable Micro Systems, UK) equipped with a 5 kg load cell. Cooked samples were dipped in cool water soon after cooking to stop the cooking process. Pasta firmness as maximum cutting force (AACC method 66-50) [24] was measured with a light knife blade (A/LKB) and a speed of 0.17 mm/s. From the force-time curve, the value of springiness was then derived. A pasta firmness/stickiness rig (HDP/PFS) at a compression speed of 0.5 mm/s and a compression force of 1 kg for 2 s was used to evaluate the stickiness (maximum peak force to separate the probe upon retraction from the sample’s surface). Ten measurements for each sample were done.

### 2.7. Thermal Properties

The thermal properties of uncooked samples were studied through differential scanning calorimetry (DSC) (DSC8000, Perkin Elmer Inc., Waltham, MA, USA). Ground samples were weighed into steel pans, distilled water was added (1:3 *w*/*w* sample:water ratio), and the pans were sealed and left at room temperature. After 20 h, samples were heated from 25 to 170 °C at a rate of 10 °C/min. The onset temperature (T_0_), the peak temperature (T_p_), the conclusion temperature (T_c_), and the gelatinization enthalpy (ΔH) were recorded using the software provided by the equipment. Results are expressed as the mean of 3 measurements for each sample.

### 2.8. In Vitro Starch Digestion of Gluten-Free Pasta

Samples (10 g) were cooked to optimum in 100 mL boiling water, drained up for 1 min, and directly analyzed. A 2-step (i.e., gastric and pancreatic phases) static in vitro starch digestion procedure was employed [25]. Cooked samples were passed through a meat mincer to mimic mastication, inserted in glass tubes, and hydrolyzed up to 180 min as detailed by Giuberti et al. [25]. Every 30 min up to 180 min liquid aliquots were taken for the measurement of the released glucose. This was done using a glucose oxidase kit (GODPOD 4058, Giesse Diagnostic snc, Rome, Italy). The area under the hydrolysis curve was measured and used to calculate the starch hydrolysis index (HI) with common white wheat bread as reference [25].

### 2.9. Sensory Analysis

The sensory profile of cooked to optimum macaroni pasta was evaluated by a 58-member panel recruited from students and staff of the Università Cattolica del Sacro Cuore (45% males and 55% females, 22–57 years old). Each member received 12 h of training prior to the test. Samples (750 g) were cooked to OCT in boiling salted water, and a cooked portion of 20 g was immediately offered to panelists. Each sample was labeled with three-digit random codes, and the order of presentation was balanced and randomized. Attributes included: color uniformity, appearance (regularity of shape, presence of deformation, cracks, and scratches), texture (hard at first chew), aroma, and taste. The test was carried out in one session, and members assigned the intensity of liking or disliking with a 9-point hedonic scale. Members were asked to comment on the overall acceptability using a 9-point hedonic scale (1–9). A score of 5 was considered as the limit of acceptability [26]. Water was provided between the evaluations. Each participant completed a written informed consent before the study.

### 2.10. Statistical Analyses

Data are presented as the mean values ± standard deviation of at least triplicate measurements. The comparison of means was conducted using the analysis of variance (One-way ANOVA) with a post hoc Tukey test at *p* < 0.05. The software IMB SPSS Statistics (Version 25) was used.

## 3. Results

### 3.1. Chemical Composition and Resistant Starch Content of Pasta

Irrespective of the annRS inclusion level, GF pasta samples were characterized by similar crude protein, crude lipid, and ash contents (Table 1).

An increase in the TDF content was measured in GF pasta added with increasing levels of annRS, the highest value recorded for 15-annRS (i.e., 9.2 g/100 g DM, *p* < 0.05). The increase in the TDF following annRS inclusion is related to the analytical procedure employed, which measures the TDF by taking into account the RS and non-digestible oligosaccharides [27,28]. The current nutritional guidelines indicate that the definition of TDF includes carbohydrate polymers that are not hydrolyzed within the human small intestine. Accordingly, the RS, being classified as a functional fiber component, should be included [29]. Previous indications reported a low dietary fiber daily intake for individuals following a GF diet [30]. Accordingly, GF foods with high dietary fiber contents can be considered beneficial [31].

The RS is a functional dietary component that helps maintain metabolic and colonic health [32,33]. In the current study, the RS was measured prior to and after the cooking step to assess the thermal behavior of the selected RS-rich ingredient. The RS content of control pasta was 0.7 g/100 g DM, confirming previous findings on similar GF food products [11]. Besides, the annRS has proven effective in increasing the RS content, with the highest values recorded for 15-annRS pasta, both in the uncooked form and after the cooking step (i.e., 7.1 and 5.8 g/100 g DM, respectively). Accordingly, an aRSr of about 80% was calculated, irrespective of the level of annRS in the formulation (Table 1). Giuberti et al. [19] reported that annRS was characterized by higher thermal stability compared to the native white sorghum starch. This suggests that starch chain interactions formed during annealing are not disrupted during gelatinization, and this restricts the accessibility of the starch chains to the starch-hydrolyzing enzymes [33,34,35]. Findings agreed with those reported on GF biscuits made with increasing levels of annRS [21]. However, it is difficult to compare present findings with the literature because this is the first time in which the annRS was used in GF pasta formulation. Indeed, some studies suggested the addition of different RS-rich ingredients in wheat-based and GF pasta. Specifically, Gelencsér et al. [16] reported that the extrusion step did not cause a significant decrease in the RS content, but, on the contrary, greater RS loss was measured after cooking (on average −50%) in wheat pasta containing two different RS-rich ingredients (i.e., high amylose starch and a phosphate starch). Foschia et al. [11], using RS from high amylose maize, attributed the 30% loss in RS during pasta making without reporting data on the cooking step. In contrast, Aravind et al. [15] did not report changes comparing uncooked and cooked wheat pasta added with RS. Recently, Bresciani et al. [8] indicated that the pasta-making, but not the cooking step, significantly decreases the RS content in high amylose enriched pasta. Differences in the experimental conditions, RS sources, and applied food preparation process could explain the disagreement between studies.

### 3.2. Pasta Quality Evaluation and In Vitro Starch Digestion

Substitution of a part of rice flour with annRS resulted in color difference on uncooked samples, but only in marginal changes after cooking (Table 2). In particular, irrespective of the annRS addition level, cooked samples exhibited lower lightness and yellowness values than the uncooked counterparts. Results were consistent with Larrosa et al. [36], which reported a decrease in L* values of GF pasta after the cooking process. Moreover, in terms of total color difference, different ΔE* values were recorded only for uncooked 10- and 15-annRS samples, being >3 when compared to the control. After cooking, all annRS containing samples exhibited ΔE* values < 3, meaning that, as perceived by the human eye, the annRS containing samples were similar in color to the control.

Different optimal cooking time was recorded among samples, varying from 9.3 min for the control to 11.6 min for 15-annRS (*p* < 0.05). Foschia et al. [11] reported longer OCT in GF pasta supplemented with an RS-rich ingredient from high amylose maize. The cooking loss represents the percentage of DM lost in the cooking water. As reported in Table 2, a decrease in the cooking loss was observed when the level of annRS in the recipe accounts from 10 to 15% *w*/*w*, thus suggesting the formation of a structure with more resistance to disintegration on boiling. This is of interest since, in GF pasta, starch polymers are less efficiently entrapped in the matrix due to the lack of gluten, thus giving a final product with generally high cooking losses [37]. These findings are consistent with Foschia et al. [11] results in which the inclusion of RS (20% *w*/*w*) to GF pasta led to a decrease in the cooking loss of about 30%. Lower values of cooking loss are considered desirable because they indicate a lower solubility of starch and a greater cooking tolerance [38]. Concerning the WAC values, higher values were recorded as the level of annRS increased in the recipe (*p* < 0.05). According to Sozer et al. [39], a longer cooking time corresponds to an increase in water absorption since more water can diffuse and interact with starch. In addition, the greater WAC of samples containing annRS can be related to the inherent characteristics of the selected RS-rich ingredient, characterized by a high WAC value and a greater ability to expose hydrophilic groups to bind water molecules [19].

Firmness, stickiness, and springiness are important attributes used to evaluate pasta quality [40]. The addition of annRS significantly increased the firmness (as maximum cutting force) of the cooked pasta, with values ranging from 1.6 N to 2.7 N for control and 15-annRS pasta, respectively (*p* < 0.05). This suggests the presence of a more compact structure following annRS inclusion in the recipe. Similar results were reported by Foschia et al. [11] in GF pasta enriched with 10–20% of RS, while Sozer et al. [39] using green banana starch as a source of RS did not report a significant effect on firmness. According to Marti and Pagani [37], the inclusion of different starch types at different levels in pasta formulation can contribute to modify the firmness of the final product to different extents due to the inherent starch characteristic, the specific starch network created during the pasta making and interactions occurring on cooking. Substitution of rice flour with annRS led to changes also in the stickiness parameter (Table 2). According to the literature, cooked pasta should have minimal stickiness values [41]. In this work, stickiness values decreased as the level of annRS increased, the lowest value recorded for 15-annRS (i.e., 1.4 N: *p* < 0.05). Results are consistent with Aravind et al. [15] and Foschia et al. [11], which reported lower stickiness values in RS-enriched pasta. According to the authors, the macromolecular reorganization induced by the RS addition could prevent excessive leaching of starch during the cooking process, preventing, in this way, stickiness and excessive cooking losses. The springiness indicates the ability of pasta to recuperate its original shape after compression. In general, GF pasta generally lacks elasticity compared to wheat of durum pasta [37]. The substitution of rice flour with annRS led to changes in springiness only at the highest level of ann-RS inclusion in the recipe (*p* < 0.05). In addition, recorded springiness values appeared in line with literature data for GF pasta but still inferior compared to gluten-containing counterparts [15,17,37].

**Figure 1:**
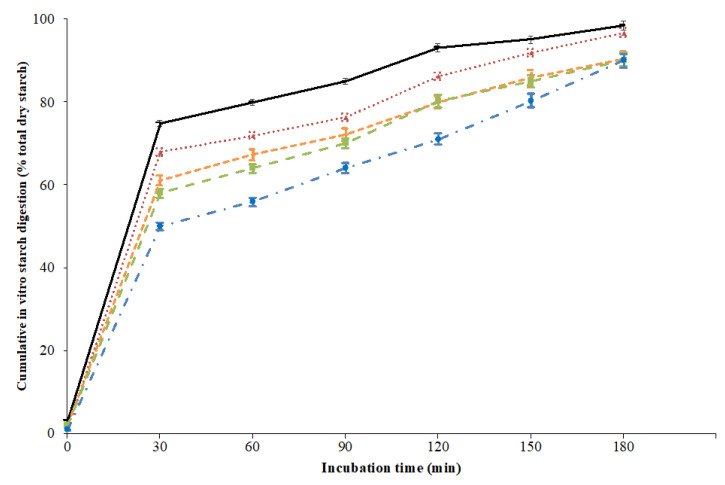
In vitro starch digestion curves of gluten-free macaroni containing resistant starch from annealed white sorghum starch (annRS). Control: gluten-free macaroni prepared with 100% *w*/*w* rice flour (red line); 5-annRS: gluten-free macaroni prepared by mixing rice flour and annRS 95:5 *w*/*w* (orange line); 10-annRS: gluten-free macaroni prepared by mixing rice flour and annRS 90:10 *w*/*w* (green line); 15-annRS: gluten-free macaroni prepared by mixing rice flour and annRS 85:15 *w*/*w* (blue line). White wheat bread is used as a reference (black line). Before analyses, pasta samples were cooked to optimal cooking time.

The in vitro starch digestion curves are presented in Figure 1.

The in vitro starch digestion curve of white wheat bread was in line with previous findings [42]. In addition, the higher RS content of pasta sample following annRS inclusion level reflected in a different extent of the in vitro starch digestibility of the cooked samples. The starch HI decreased significantly (*p* < 0.05) as the level of substitution of annRS increased, with values ranging from 91 for control pasta to about 73 for 15-annRS. This can be related to the lower susceptibility of annRS to enzymatic digestion. In cereal products, the RS fraction is not digestible neither in vitro nor in vivo; consequently, RS does not contribute to the release of glucose during the enzyme hydrolysis, which leads to a decrease in the starch HI [33,43]. Similar results have been reported for GF biscuits [21]. In addition, the possible role of the product’s hardness could also partially preserve the starch’s granular structural integrity during cooking and/or modulate the in vitro accessibility of enzymes to starch. This might have contributed towards the reduction in the starch HI of the samples, in line with previous indications [44,45].

### 3.3. Thermal Properties

The thermal properties of GF pasta samples are presented in Table 3. Control pasta was characterized by T_0_, T_p_, T_c_, and ΔH mean values of 60.3 °C, 68.2 °C, 74.4 °C, and 3.2 J/g. Marti et al. [38] reported that 100% rice pasta made with a conventional extrusion process exhibited a peak in the range of 55.4–72.5 °C, in line with the current findings. The data obtained by DCS suggested that 10-annRS and 15-annRS samples required higher temperature values for melting (from 68.3 to 86.2 °C and from 73.1 to 89.1 °C, respectively) with respect to 5-annRS and control pasta, thus resulting in a GF pasta more stable during heating. In addition, both 10-annRS and 15-annRS pasta required more energy for gelatinization (on average 5 J/g) than the other samples. Taken together, present DSC findings indicated greater thermal stability of GF pasta formulated by replacing rice flour with at least 10 g/100 g (*w*/*w*) of ann-RS. These results are consistent with the pasta behavior on cooking: the strong network obtained following annRS addition at greater inclusion level in the recipe may contribute to explain the lower cooking loss value reported for 10-annRS and 15-ann-RS pasta (Table 2), in line with previous findings [38]. In addition, reported differences in the starch gelatinization properties among samples might be related to the thermal properties of annRS and to possible differences in the starch organization/architecture following annRS inclusion during the pasta-making process. In particular, Giuberti et al. [19] reported that annealed white sorghum starch was characterized by the greatest ΔH values (i.e., 14.6 J/g), along with the greater gelatinization transition temperatures when compared to the native counterpart.

### 3.4. Sensory Analysis

The mean values for each sensorial attribute of control and annRS enriched pasta samples are presented in Table 4.

No significant difference was observed among samples in color, aroma, or taste, with average values of 5.4, 4.6, and 5.1, respectively, thus indicating that the type of RS, along with its relative amount in the recipe, did not cause changes in these parameters according to the sensory panel. This is probably related to the neutral flavor of the annRS ingredient [21,22,23,24,25,26,27,28,29,30,31,32,33,34,35,36,37,38,39,40,41,42,43]. These findings appear consistent with Gelencsér et al. [46], which reported no differences between pasta enriched with RS and control wheat-based pasta. Sensory scores for color attributes appeared in line with the instrumental values in which color differences between cooked control pasta and annRS enriched pasta were not detected. In addition, the texture of 10- and 15-annRS were relatively more appreciated by panelists with respect to the other pasta samples, thus confirming the effect on texture measured by the instrumental analysis. The appearance attribute showed the lowest score for the 15-annRS sample (5.3; *p* < 0.05), and a significant decrease in the overall acceptance was measured as the level of annRS increased in the formulation. However, all samples resulted in a score higher than 5 for the overall acceptance value, which is considered as the limit of acceptability [26]. Taken together, present data shows that the addition of annRS to pasta up to 15 g/100g *w*/*w* has a minimal effect on the sensory attributes, in line with previous findings [15,46].

## 4. Conclusions

The annRS has potential application as a value-added ingredient to produce GF pasta with high RS content and lower in vitro starch digestion with respect to 100% rice counterpart. The substitution of common rice flour with 15 g/100 g *w*/*w* of annRS also allows using the “high in fiber” claim [47]. Blending rice flour with increasing levels of annRS resulted in longer optimal cooking time, lower cooking losses, along with positive changes in texture and stickiness, thus suggesting the formation of a structure with more resistance to boiling. However, the lightness of uncooked pasta decreased as the level of annRS increased in the recipe, which may potentially reduce the attractiveness of the new formulated GF pasta to consumers. Sensory attributes were only marginal affected by the annRS inclusion. Present findings underline the suitability of this RS ingredient in GF pasta production up to 15 g/100 g *w*/*w*. Further studies to assess the in vivo digestibility and potential health benefits are desirable.

## Figures and Tables

**Table 1:** Chemical composition (g/100 g dry matter) and apparent resistant starch retention (aRSr, %) of gluten-free macaroni containing resistant starch (RS) from annealed white sorghum starch (annRS).

	Gluten-Free Pasta			
	Control ^1^	5-annRS ^2^	10-annRS ^3^	15-annRS ^4^
Moisture (g/100 g)	11.3 ± 0.33 ^a^	10.9 ± 0.98 ^a^	11.1 ± 0.08 ^a^	11.8 ± 0.77 ^a^
Total starch	87.6 ± 2.11 ^a^	86.4± 1.91 ^a^	84.3 ± 2.11 ^b^	80.1 ± 2.13 ^c^
Crude protein	8.0 ± 0.23 ^a^	8.2 ± 0.55 ^a^	7.9 ± 0.33 ^a^	7.8 ± 0.77 ^a^
Crude lipid	1.1 ± 0.11 ^a^	1.3 ± 0.12 ^a^	1.2 ± 0.09 ^a^	1.2 ± 0.10 ^a^
Ash	0.5 ± 0.01 ^a^	0.4 ± 0.01 ^a^	0.4 ± 0.02 ^a^	0.3 ± 0.03 ^a^
Dietary fiber	1.2 ± 0.12 ^a^	4.0 ± 0.89 ^b^	5.7 ± 0.72 ^c^	9.2 ± 1.11 ^d^
RS (uncooked)	0.7 ± 0.04 ^a^	2.6 ± 0.12 ^b^	4.3 ± 0.55 ^c^	7.1 ± 0.82 ^d^
RS (cooked to optimum)	0.04 ± 0.01 ^a^	2.1 ± 0.03 ^b^	3.6 ± 0.22 ^c^	5.8 ± 0.59 ^d^
aRSr (%)	5.7 ± 0.66	80.1 ± 2.33	83.2 ± 3.11	81.4 ± 3.27

Means in the same line with different superscript differed at *p* < 0.05. ^1^ Gluten-free macaroni prepared with 100% *w*/*w* rice flour. ^2^ Gluten-free macaroni prepared by mixing rice flour and annRS 95:5 *w*/*w*. ^3^ Gluten-free macaroni prepared by mixing rice flour and annRS 90:10 *w*/*w*. ^4^ Gluten-free macaroni prepared by mixing rice flour and annRS 85:15 *w*/*w*.

**Table 2:** Quality parameters, texture analysis, and in vitro starch hydrolysis index of gluten-free macaroni containing resistant starch from annealed white sorghum starch (annRS).

	Gluten-Free Pasta			
	Control ^1^	5-annRS ^2^	10-annRS ^3^	15-annRS ^4^
Lightness L* (uncooked)	94.5 ± 0.16 ^c^	93.2 ± 0.02 ^c^	91.2 ± 0.13 ^b^	89.1 ± 0.17 ^a^
Redness a* (uncooked)	−0.3 ± 0.01 ^a^	−0.4 ± 0.01 ^a^	0.2 ± 0.02 ^b^	0.4 ± 0.01 ^b^
Yellowness b* (uncooked)	5.3 ± 0.05 ^a^	5.0 ± 0.04 ^a^	5.1 ± 0.72 ^a^	5.2 ± 0.11 ^a^
ΔE* (uncooked)	-	1.3	4.3	5.4
Lightness L* (cooked to optimum)	90.8 ± 0.22 ^a^	89.8 ± 0.11 ^a^	88.9 ± 0.20 ^a^	88.4 ± 0.15 ^a^
Redness a*(cooked to optimum)	−0.4 ± 0.01 ^a^	−0.3 ±0.01 ^a^	−0.3 ±0.02 ^a^	−0.4 ± 0.01 ^a^
Yellowness b* (cooked to optimum)	4.5 ± 0.05 ^b^	3.3 ± 0.03 ^a^	3.1 ± 0.02 ^a^	2.8 ± 0.01 ^a^
ΔE* (cooked to optimum)	-	1.6	2.4	2.9
Optimal cooking time (min)	9.3 ± 0.17 ^a^	9.6 ± 0.22 ^a^	10.6 ± 0.12 ^b^	11.6 ± 0.34 ^b^
Cooking loss (%)	12.2 ± 0.46 ^a^	12.1 ± 0.33 ^a^	10.4 ± 0.70 ^b^	10.1 ± 0.27 ^b^
Water absorption capacity (%)	101.3 ± 3.12 ^a^	104.9 ± 2.22 ^b^	107.2 ± 4.00 ^c^	109.1 ± 3.51 ^c^
Firmness (N)	1.6 ± 0.08 ^a^	2.1 ± 0.10 ^b^	2.3 ± 0.04 ^b^	2.7 ± 0.11 ^c^
Stickiness (N)	2.7 ± 0.21 ^b^	2.6 ± 0.14 ^b^	1.7 ± 0.15 ^a^	1.4 ± 0.08 ^a^
Springiness	0.44 ± 0.11 ^a^	0.42 ± 0.09 ^a^	0.44 ± 0.11 ^a^	0.56 ± 0.12 ^b^
In vitro starch hydrolysis index ^5^	91.0 ± 3.12 ^d^	83.2 ± 2.04 ^b^	80.2 ± 3.01 b	73.1 ± 2.16 ^a^

Means in the same line with different superscript differed at *p* < 0.05. ^1^ Gluten-free macaroni prepared with 100% *w*/*w* rice flour. ^2^ Gluten-free macaroni prepared by mixing rice flour and annRS 95:5 *w*/*w*. ^3^ Gluten-free macaroni prepared by mixing rice flour and annRS 90:10 *w*/*w*. ^4^ Gluten-free macaroni prepared by mixing rice flour and annRS 85:15 *w*/*w*. ^5^ Calculated using white wheat bread as reference (HI = 100 by definition).

**Table 3:** Thermal properties of gluten-free macaroni containing resistant starch from annealed white sorghum starch (annRS).

	Gluten-Free Pasta			
	Control ^1^	5-annRS ^2^	10-annRS ^3^	15-annRS ^4^
Onset temperature T_0_ (°C)	60.3 ± 2.16 ^a^	62.1 ± 1.43 ^a^	68.3 ± 1.93 ^b^	73.1 ± 2.33 ^c^
Peak temperature T_p_ (°C)	68.2 ± 1.55 ^a^	70.3 ± 0.94 ^a^	81.2 ± 2.02 ^b^	84.7 ± 1.01 ^c^
Conclusion temperature T_c_ (°C)	74.4 ± 1.02 ^a^	77.3 ± 0.04 ^a^	86.2 ± 0.82 ^b^	89.1 ± 0.61 ^b^
Gelatinization enthalpy ΔH (J/g)	3.2 ± 0.17 ^a^	3.4 ± 0.34 ^a^	4.8 ± 0.23 ^b^	5.2 ± 0.14 ^b^

Means in the same line with different superscript differed at *p* < 0.05. ^1^ Gluten-free macaroni prepared with 100% *w*/*w* rice flour. ^2^ Gluten-free macaroni prepared by mixing rice flour and annRS 95:5 *w*/*w*. ^3^ Gluten-free macaroni prepared by mixing rice flour and annRS 90:10 *w*/*w*. ^4^ Gluten-free macaroni prepared by mixing rice flour and annRS 85:15 *w*/*w*.

**Table 4:** Average sensory scores of gluten-free macaroni containing resistant starch from annealed white sorghum starch (annRS).

	Gluten-Free Pasta			
	Control ^1^	5-annRS ^2^	10-annRS ^3^	15-annRS ^4^
Color	5.4 ± 0.56 ^a^	5.5 ± 0.12 ^a^	5.3 ± 0.34 ^a^	5.3 ± 0.45 ^a^
Appearance	6.1 ± 0.32 ^a^	6.1 ± 0.54 ^a^	5.8 ± 0.61 ^a^	5.3 ± 0.31 ^b^
Texture	4.6 ± 0.15 ^a^	4.8 ± 0.32 ^a^	5.2 ± 0.72 ^b^	5.9 ± 0.22 ^c^
Aroma	5.4 ± 0.32 ^a^	5.5 ± 0.43 ^a^	5.3 ± 0.11 ^a^	5.3 ± 0.27 ^a^
Taste	5.0 ± 0.41 ^a^	5.0 ± 0.33 ^a^	5.1 ± 0.31 ^a^	5.1 ± 0.66^a^
Overall acceptance	6.3 ± 0.32 ^b^	6.1 ± 0.14 ^b^	5.4 ± 3.01 ^a^	5.3 ± 2.16 ^a^

Means in the same line with different superscript differed at *p* < 0.05. ^1^ Gluten-free macaroni prepared with 100% *w*/*w* rice flour. ^2^ Gluten-free macaroni prepared by mixing rice flour and annRS 95:5 *w*/*w*. ^3^ Gluten-free macaroni prepared by mixing rice flour and annRS 90:10 *w*/*w*. ^4^ Gluten-free macaroni prepared by mixing rice flour and annRS 85:15 *w*/*w*.
